# Exploring the relationship between pyroptosis and inflammatory bone loss: Evidence from a cigarette smoke-induced osteoporosis mouse model

**DOI:** 10.1016/j.heliyon.2024.e35715

**Published:** 2024-08-05

**Authors:** Guang Wang, Hongming Li, Xinyue Hu, Yiyi Wang, Guoqiang Zhu, Hongliang Zhou, Zilin Liang, Zhenxing Wang, Andreas Nuessler, Zhangyuan Lin, Hui Xie, Sheng Zhu

**Affiliations:** a Department of Orthopedics, Xiangya Hospital Central South University, Changsha, Hunan, 410008, China; b Movement System Injury and Repair Research Center, Xiangya Hospital Central South University, Changsha, Hunan, 410008, China; c National Clinical Research Center for Geriatric Disorders, Xiangya Hospital Central South University, Changsha, Hunan, 410008, China; d Department of Trauma and Reconstructive Surgery, Siegfried Weller Institute for Trauma Research, Eberhard Karls University Tuebingen, BG Trauma Center Tuebingen, 72076, Tuebingen, Germany

**Keywords:** Cigarette smoking, Osteoporosis, Pyroptosis, BMSCs

## Abstract

Smoking is by far one of the greatest public health threats and is recognized as an important predisposing factor for osteoporosis. Exposure to cigarette smoke (CS) has been reported to be associated with inflammation-associated diseases through the induction of pyroptosis. Nevertheless, the correlation between pyroptosis and bone loss induced by CS remains uninvestigated. Here, a mouse model of mainstream smoke exposure-induced osteoporosis was established. μCT, biomechanical testing, and immunohistochemical staining of bone tissue were used to assess the deleterious effects of CS on bone metabolism. *In vitro*, the effects of cigarette smoke extracts (CSE) on mouse primary bone marrow-derived mesenchymal stem cells (BMSCs) were tested by cell viability assays, gene and protein expression assays, and alizarin red staining. The utilization of the pyroptosis inhibitor MCC950 served to confirm the critical role of BMSCs pyroptosis in CS-induced osteoporosis. Our results indicated that exposure to mainstream smoke led to a notable decrease in the quantity of osteoblasts and hindered the process of osteogenic differentiation in mice. Additionally, there was a significant increase in the expression of pyroptosis-related proteins in the bone marrow. The inhibitory effects of CSE on cell viability and osteogenic differentiation of BMSCs were found to be dose-dependent *in vitro*. However, the presence of the pyroptosis inhibitor MCC950 significantly improved the impaired osteogenic differentiation and bone mineralization caused by CSE. These results highlight the crucial involvement of BMSCs pyroptosis in the development of bone loss induced by CS. In summary, the findings of this study provide novel evidence that CS exerts a detrimental effect on the process of osteogenesis in BMSCs through the induction of pyroptosis, ultimately leading to bone loss. Inhibition of pyroptosis effectively attenuated the toxicological effects of CS on BMSCs, providing a new target for preventing inflammatory osteoporosis.

## 1 Introduction

The tobacco epidemic is by far one of the greatest public health threats facing the world, causing more than 8 million deaths each year [1]. Cigarette smoke (CS) is a toxic mixture containing 7000 chemical substances, and most of which are considered to be pathogenic or carcinogenic to humans [2]. Long-term CS exposure is known to contribute to the development of respiratory diseases, cardiovascular diseases and malignancies [3]. In the skeletal system, CS has long been recognized as an important predisposing factor for osteoporosis and osteoporotic fractures [4]. In the skeletal system, CS has long been recognized as an important predisposing factor for osteoporosis and osteoporotic fractures [5,6]. Osteoporosis leads to reduced quality of life, disability adjusted lifespan, and imposes a heavy financial burden on the health insurance systems of the countries responsible for caring for such patients [7]. Therefore, it is of great clinical and practical importance to understand the specific mechanisms of CS-induced bone loss [8].

The precise mechanisms through which smoking induces osteoporosis remain uncertain, with the absence of standardized animal exposure models constituting a notable constraint [9]. CS acts as a chronic “inflammatory stimulus” that induces the cellular secretion of a variety of pro-inflammatory factors, leading to a systemic inflammatory response and inflammation-related diseases [10]. The consequences of unchecked CS-induced inflammation and immune dysregulation continue to be an area of active research.

Pyroptosis is a novel mode of programmed cell death that is closely associated with inflammatory responses and which can be induced by a variety of pathogens and external stimuli [11]. The canonical molecular pathway is the activation of caspase-1 by the cellular inflammasome NLRP3 upon sensing inflammatory stimuli which in turn cleaves GasderminD (GSDMD), ultimately leading to cell death and the release of activated IL-1β and IL-18 into the extracellular environment [12]. Recent studies have demonstrated the correlation between pyroptosis and various inflammatory and age-related ailments [13,14]. In the realm of bone diseases, research has indicated a potential correlation between pyroptosis and both disc degeneration and spinal cord injury [15,16]. In vivo studies suggest that the suppression of pyroptosis may offer relief for rheumatoid arthritis and traumatic osteomyelitis [17,18]. To date, there is a lack of empirical evidence supporting a correlation between pyroptosis and osteoporosis, but heightened levels of NLRP3 have been detected in a murine model of menopausal osteoporosis [19].

Collectively, pyroptosis in bone cells could present a viable hypothesis elucidating the underlying mechanism of inflammation-related osteoporosis. In this study, we aim to establish a feasible animal model of CS-induced osteoporosis, elucidate the impact of CS exposure on bone metabolism in both *in vivo* and *in vitro* settings, and innovatively investigate the involvement of pyroptosis in mediating CS-induced osteoporosis.

## 2 Methods

### 2.1 Animals and CS exposure

The Ethical Review Board at Xiangya Hospital of Central South University approved the experimental procedures and animal care. In this study, male C57BL/6 mice aged 6 weeks were used. An animal mainstream smoke exposure device was designed and applied to CS exposure for experimental Mice. Standardized commercial cigarettes (Baisha cigarettes with filter, Hunan, China; tar 11 mg, nicotine 0.9 mg, CO 12 mg) were used in this study. The partial pressure of oxygen is maintained at 19%–20 % and carbon monoxide at 300 ppm ± 60 ppm in the exposure chamber (1 h each time, twice a day, and six days a week of exposure) [20]. A real-time air composition monitor was used to measure air parameters in the exposure chamber.

### 2.2 Preparation of cigarette smoke extracts (CSE)

According to our previous work [21], a standard negative pressure pump was used to create a negative pressure gas stream. We collected smoke and dissolved it into cell culture medium through a standard gas wash bottle, calibrated with a microplate reader (Varioskan LUX Multi-mode, Thermo Fisher Scientific, Waltham, USA), and filtered and diluted into different concentrations of CSE solution for cell intervention. In general, 10 % CSE stands for smoking 20 cigarettes per day [22].

### 2.3 Microcomputed tomography (μCT) analysis

The femora dissected from mice were fixed in 4 % paraformaldehyde overnight and examined using high-resolution μCT (Skyscan 1176) as previously described [23]. In this scanner, the voltage was 50 kV, the current was 400A, and the resolution was 11.4 μm per pixel. The femoral parameters were analyzed using image reconstruction software (NRecon), data analysis software (CTAn v1.11), and three-dimensional model visualization software (CTVol v2.2).

### 2.4 Three-point bending test

Three-point bending tests were conducted on a mechanical testing machine (Instron 3343, Canton, USA) to assess bone strength as describe previously [24]. In short, femoral tissue samples were placed on a lower support bar at a distance of 8 mm between the pivot points. The ultimate load (N) was calculated based on load-deflection curves generated from vertical compression loads applied at a constant rate of 5 mm per minute until fracture occurred.

### 2.5 Transcriptome RNA sequencing processing and analysis

We conducted transcriptome RNA sequencing on femoral tissues of mice exposed to CS for 8 weeks. Transcriptome RNA sequencing was performed in three biological replicates for each group. As per manufacturer's instructions (Illumina, San Diego, USA), RNA extraction, purification, reverse transcription, library construction, and sequence were performed at Shanghai Majorbio Bio-pharm Biotechnology (Shanghai, China). Data analysis was performed on Majorbio Cloud Platform (http://www.majorbio.com). Osteogenesis-related genes were cited on GeneCards website (https://www.genecards.org).

### 2.6 Cell culture

As described previously, wild-type C57BL/6 mice femurs and tibias were collected, and primary bone marrow mesenchymal stem cells (BMSCs) were isolated [25]. We cultured BMSCs with 10 % fetal bovine serum (FBS; Cat. No. 12664025; Gibco, Grand Island, USA). Then 1 % penicillin-streptomycin was added (Cat. No. P1400; Solarbio, Beijing, China). A humidified environment of 37 °C and 5 % CO2 was used to culture BMSCs.

### 2.7 Calcein-AM and propidium iodide (PI) assays

BMSCs viability was assessed using a Calcein-AM/PI double staining kit (Yeasen, China) according to the manufacturer’ s instructions. Briefly, following treatment with different concentrations of CSE for 24 h, 5 μl of 2 mM Calcein-AM and 15 μl of 1.5 mM PI were diluted in 1 mL of assay buffer and applied to NRVMs. A fluorescence microscope (Leica DMI6000B, Solms, Germany) was used to acquire images after 30 min of staining. Image-Pro Plus 6 software was used for data acquisition.

### 2.8 Cell proliferation assay

The BMSCs were cultured in DMEM medium with different concentrations of CSE or PBS in 96-well culture plates. After 24 h, BMSCs were cultured for an additional 3 h in fresh complete medium with Cell Counting Kit-8 reagent (7 Sea Biotech, Shanghai, China). In this experiment, the absorbance at 450 nm was measured using a microplate reader (Varioskan LUX Multi-mode, Thermo Fisher Scientific, Waltham, USA).

### 2.9 Osteogenic differentiation assay

The BMSCs were seeded in 48-well plates and cultured in osteogenic medium (Cat. No. MUCMX90021; Cyagen Biosciences Inc, Guangzhou, China) after 80 % confluence was reached. BMSCs cultured in α-MEM with 10 % FBS were used as a negative control. Every other day, half the medium was replaced. Following osteogenic differentiation for 14 days, the BMSCs were stained with Alizarin Red S (ARS) (Solarbio, G1452, Beijing, China). Image-Pro Plus 6 was used to analyze the ARS-positive area.

### 2.10 Tissue staining assays

The femurs were fixed in 4 % paraformaldehyde for 48 h, then decalcified in 0.5 m EDTA (pH = 7.4) with constant shaking at 4 °C for approximately 3 days. And then, a graded concentration of ethanol was used to dehydrate the samples, then paraffin was applied to embed them. Immunohistochemical staining for OCN, NLRP3, CASPASE-1 and IL-1β as previously described [26]. The antibodies are listed in Table 1. The images were acquired with an Olympus CX31 optical microscope (Olympus, Tokyo, Japan). The datas were measured by using Image-Pro Plus 6 software.

**Table 1:** Antibodies used for Immunohistochemical staining and Western blot.

**Name**	**Cat.No**	**Brand**	**Concentration(IHC)**	**Concentration(WB)**
GAPDH	10494-1-AP	Proteintech	Na	1: 5000
OCN	gb11233	Servicebio	1: 200	Na
NLRP3	68102-1-Ig	Proteintech	1: 200	1: 2000
CASPASE-1	K107599P	Solarbio	1: 100	1: 1000
GSDMD	ab209845	Abcam	Na	1: 1000
IL-1β	26048-1-AP	Proteintech	1: 200	1: 1000
HRP conjugated Goat Anti-Rabbit lgG(H + L)	gb23303	Servicebio	1: 200	1: 1000
HRP conjugated Goat Anti-Mouse lgG(H + L)	gb23301	Servicebio	1: 200	1: 1000

“Na” stands for “not applicable".

### 2.11 qRT-PCR analysis

Total RNA was extracted from both cultured cells and femur tissues using TRIzol reagent (Invitrogen). Following the assessment of RNA purity and concentration, reverse transcription to complementary DNA (cDNA) was carried out using the PrimeScript RT kit (Takara). Quantitative real-time polymerase chain reaction (qRT-PCR) was performed employing the TB Green Premix Ex *Taq*II (Takara). In this investigation, glyceraldehyde 3-phosphate dehydrogenase (Gapdh) served as the reference gene. The determination of relative mRNA expression levels was achieved using the 2^−ΔΔCT method. Detailed primer sequences can be found in Table 2.

**Table 2:** Primers used for qRT-PCR.

**gene**	**Primer sequences(5′-3′)**	
Gapdh	forward	TGGATTTGGACGCATTGGTC
	reverse	TTTGCACTGGTACGTGTTGAT
Nlrp3	forward	ATTACCCGCCCGAGAAAGG
	reverse	TCGCAGCAAAGATCCACACAG
Caspase-1	forward	ACAAGGCACGGGACCTATG
	reverse	TCCCAGTCAGTCCTGGAAATG
Gsdmd	forward	CCATCGGCCTTTGAGAAAGTG
	reverse	ACACATGAATAACGGGGTTTCC
IL-1β	forward	GAAATGCCACCTTTTGACAGTG
	reverse	TGGATGCTCTCATCAGGACAG
Bglap	forward	CTTGGTGCACACCTAGCAGA
	reverse	CTCCCTCATGTGTTGTCCCT
Runx2	forward	GACTGTGGTTACCGTCATGGC
	reverse	ACTTGGTTTTTCATAACAGCGGA
Alp	forward	CCAACTCTTTTGTGCCAGAGA
	reverse	GGCTACATTGGTGTTGAGCTTTT
Col1a1	forward	GCTCCTCTTAGGGGCCACT
	reverse	CCACGTCTCACCATTGGGG

### 2.12 Western blotting analysis

Total proteins were extracted from BMSCs using RIPA buffer (Beyotime) supplemented with a protease inhibitor cocktail (Sigma). The extracted proteins underwent separation through sodium dodecyl sulfate-polyacrylamide gel electrophoresis (SDS-PAGE) and subsequent transfer to polyvinylidene fluoride membranes (Millipore). Following a 2-h blocking step with 5 % non-fat milk, the membranes were incubated overnight at 4 °C with primary anti-bodies. And then, the membranes were incubated with horseradish peroxidase (HRP)-conjugated secondary anti-bodies at 37 °C for 1h at room temperature. The antibodies are listed in Table 1. Immunoreactive bands were visualized utilizing a chemiluminescence reagent (Thermo Fisher Scientific, Waltham, USA) and imaged using the ChemiDoc XRS with Image Lab Software (Bio–Rad, California, USA). Quantification of the Western blot bands was performed using ImageJ software.

### 2.13 Statistical analysis

Statistical analyses were performed using GraphPad Prism 9 software. Unpaired, two-tailed Student's t-test was conducted for comparisons between two groups. Ordinary one-way ANOVA test was used for comparisons among multiple groups. Statistical significance was defined as *p < 0.05, **p < 0.01, ***p < 0.001, and ****p < 0.0001, indicating the level of significance. All experiments were repeated in triplicate or more to confirm the results unless otherwise stated.

## 3 Results

### 3.1 Establishment of a standardized CS-induced osteoporosis mouse model

**Fig. 1:**
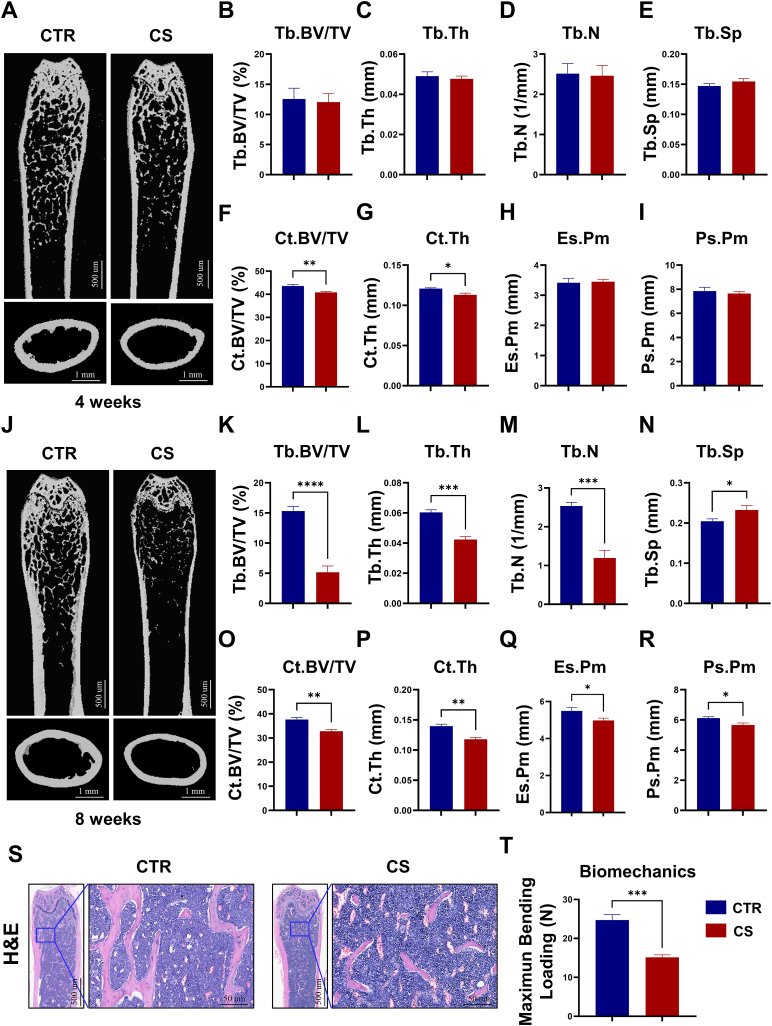
CS exposure caused osteoporotic alterations in mice. **(A)** Representative μCT-reconstructed images of femora from mice in control (CTR) and CS groups (4 week-exposure), scale bars: 500 μm (Above) and 1 mm (bottom). **(B–I)** Quantitative analysis of trabecular bone volume fraction (Tb. BV/TV), trabecular thickness (Tb. Th), trabecular number (Tb. N), trabecular separation (Tb. Sp), cortical bone volume fraction (Ct. BV/TV), cortical thickness (Ct. Th), endocortical perimeter (Es. *Pm*), periosteal perimeter (Ps. *Pm*), n = 6–10. **(J)** Representative μCT-reconstructed images of femora from mice in CTR and CS groups (8 week-exposure), scale bars: 500 μm (Above) and 1 mm (bottom). **(K–R)** Quantitative analysis of Tb. BV/TV, Tb. Th, Tb. N, Tb. Sp, Ct. BV/TV, Ct. Th, Es. *Pm*, Ps. *Pm*, n = 5. **(S)** Representative H&E staining images of femora from mice in CTR and CS groups (8 week-exposure), scale bars: 500um (Left), 50 μm (Right), n = 3. **(T)** Three-point bending measurement of femur ultimate load in CTR and CS groups (8 week-exposure), n = 6. *p < 0.05, **p < 0.01, ***p < 0.001, or ****p < 0.0001.

In light of the absence of a universally accepted animal model for simulating CS-induced osteoporosis, we designed a specialized apparatus intended for exposing animals to mainstream smoke (Supplemental Fig. S1). Young C57BL/6 mice were subjected to cigarette smoke (CS) in a controlled air environment, where the concentration of carbon monoxide (CO) was standardized. The results obtained from μCT analysis after a duration of four weeks indicated a propensity towards bone loss in the femur of CS-exposed mice, but the difference was not statistically significant (Fig. 1A–I). A notable decline in bone density was observed in mice exposed to CS for a duration of 8 weeks, as evidenced by significantly reduced trabecular bone volume fraction (Tb. BV/TV), trabecular number (Tb. N), trabecular thickness (Tb. Th), cortical bone area fraction (Ct. BV/TV), cortical thickness (Ct. Th), periosteal perimeter (Ps. *Pm*) and endocortical perimeter (Es. *Pm*) and increased trabecular separation (Tb. Sp) (Fig. 1J–R). In addition, the analysis of H&E staining demonstrated a notable decrease in the quantity of femoral trabeculae in the CS group in comparison to the control (CTR) group. (Fig. 1S). Furthermore, the three-point bending test revealed that exposure to CS resulted in a significant reduction in the maximum bending load of the femur, indicating a detrimental impact on bone strength (Fig. 1T). Thus, a feasible mouse model of CS-induced osteoporosis was established by 8-week CS exposure and will be used in the following *in vivo* experiments.

### 3.2 Exposure to CS leads to a decrease in the quantity of osteoblasts and hinders the process of osteogenic differentiation

**Fig. 2:**
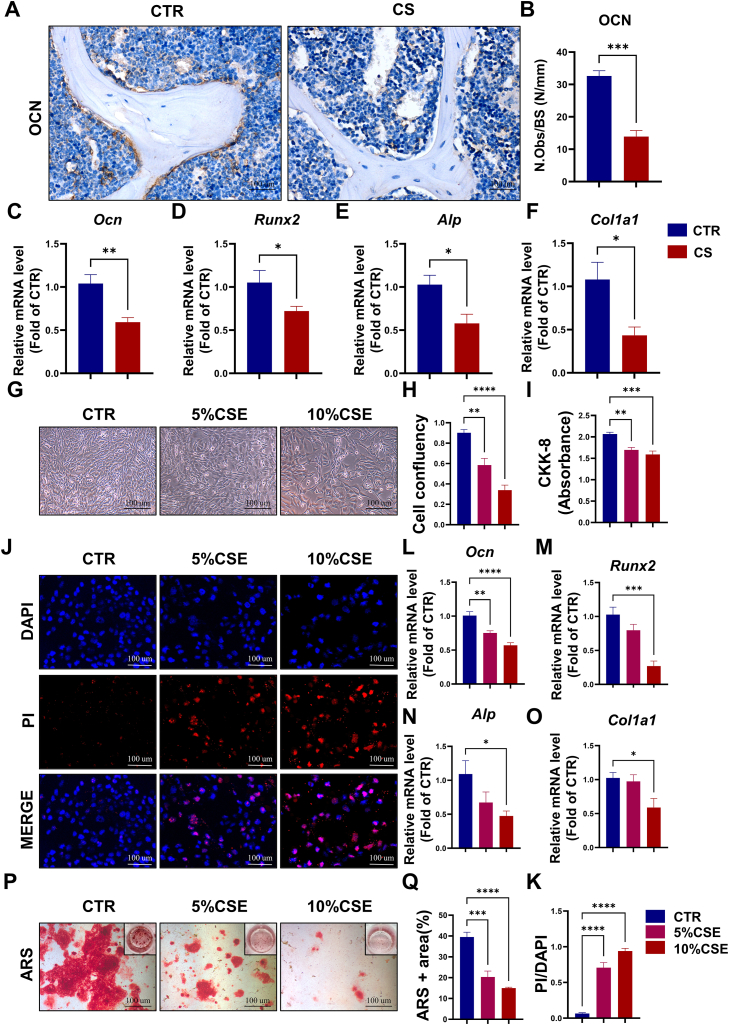
Exposure to CS leads to a decrease in the quantity of osteoblasts and hinders the process of osteogenic differentiation. **(A)** Representative OCN-stained sections of femora from mice in CTR and CS groups, scale bars: 100 μm. **(B)** Quantification of osteoblast number per bone surface (N. OBs/BS), n = 3–4. **(C–F)** The mRNA expression of osteogenesis-related genes (*Ocn*, *Runx2*, *Alp* and *Col1a1*) of femora from mice in CTR and CS groups. **(G)** Representative images of BMSCs microscopy in CTR, 5 % CSE and 10 % CSE groups, scale bars: 100 μm. **(H)** statistical analysis of cell confluency, n = 6. **(I)** Cell Counting Kit-8 (CCK-8) assay. n = 6. **(J)** Representative fluorescent staining images of BMSCs death in CTR, 5 % CSE and 10 % CSE groups, scale bars: 100 μm. **(K)** Statistical analysis of PI/DAPI, n = 9. **(L**–**O)** The mRNA expression of osteogenesis-related genes (*Ocn*, *Runx2*, *Alp* and *Col1a1*) from the BMSCs in CTR, 5 % CSE and 10 % CSE groups. **(P)** Representative ARS staining images of BMSCs microscopy in CTR, 5 % CSE and 10 % CSE groups, scale bars: 100um. **(Q)** Statistical analysis of ARS + area. n = 3–4 *p < 0.05, **p < 0.01, ***p < 0.001, or ****p < 0.0001.

Osteoporosis denotes a dysregulation in the dynamic bone remodeling process, characterized by either an overactive osteoclast-mediated bone resorption or insufficient osteoblast-mediated bone formation. OCN is a marker of osteoblast differentiation. Immunohistochemical staining of OCN showed that osteoblast numbers were significantly decreased in the CS group compared with control group (Fig. 2A and B). The qRT-PCR results showed that CS exposure led to a significant reduction in the expression of osteogenesis-related genes (*Ocn*, *Runx2*, *Alp* and *Col1a1*) in the CS group (Fig. 2C–F). To further clarify the effect of CS exposure on the osteogenic differentiation process, we used different concentrations of CSE to treat mouse primary BMSCs *in vitro*. We found that CSE leads to a significant decrease in the viability of BMSCs in a dose-dependent manner according to cell morphology (Fig. 2G and H), propidium iodide (PI) staining (Fig. 2J and K) and the CCK-8 assay (Fig. 2I). The qRT- PCR results showed that the expression of osteogenesis-related genes (*Ocn*, *Runx2*, *Alp* and *Col1a1*) declined with increasing CSE concentrations (Fig. 2L–O). Meanwhile, the Alizarin-red staining (ARS) results showed that CSE significantly reduced matrix mineralization of differentiated BMSCs (Fig. 2P and Q), suggesting CSE impaired the process of bone formation.

### 3.3 CS potentially impairs bone metabolism by inducing pyroptosis based on transcriptomic analyses

**Fig. 3:**
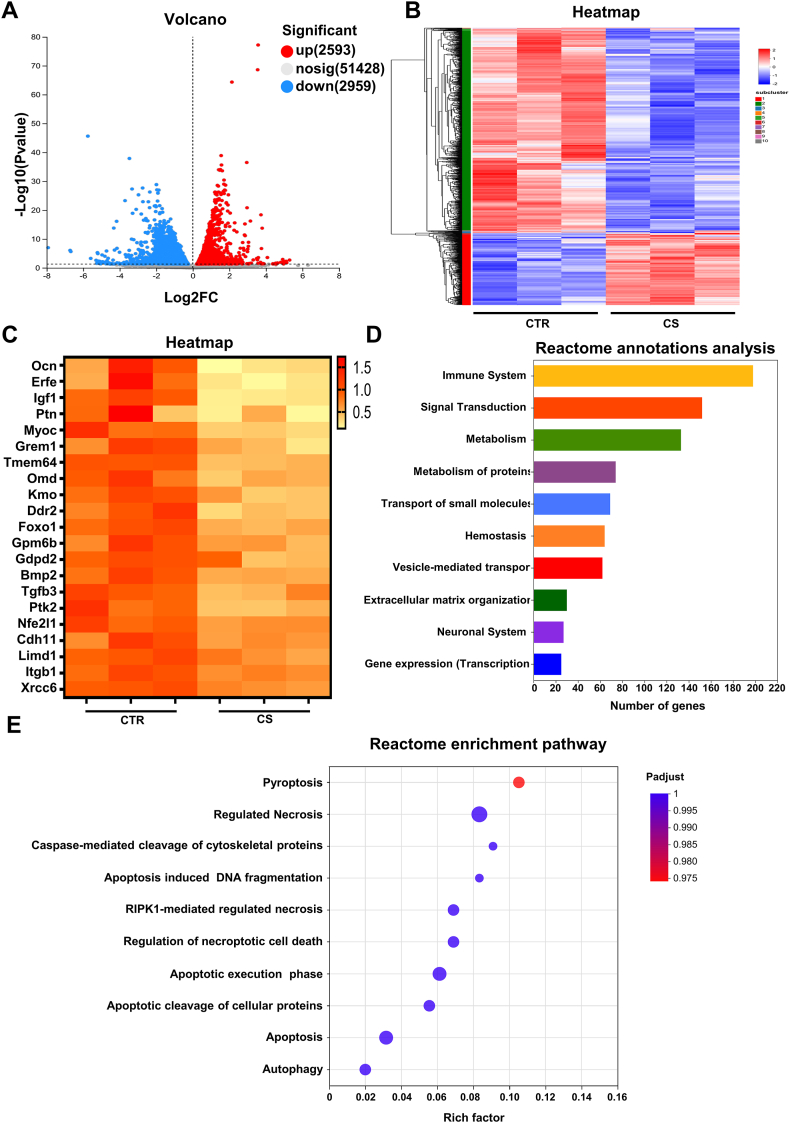
CS potentially impairs bone metabolism by inducing pyroptosis based on transcriptomic analyses. **(A)** Volcano plots showed genes that were significantly up-regulated (red dots) and down-regulated (blue dots) in the bone tissue of mice in the CS group (exposed for 8 weeks) compared to the control group. **(B)** Heatmap showed differential genes of bone tissue in CS-exposed mice conpared to controls. **(C)** Heatmap showed osteogenesis-related genes of bone tissue between CS-exposed mice and controls. **(D)** Histogram showed the top 10 enriched pathways of differential genes of bone tissue in CS-exposed mice identified by reactome annotations analysis. **(E)** Bubble diagrams showed the enrichment levels of differential genes in pathways related to programmed cell death. (For interpretation of the references to colour in this figure legend, the reader is referred to the Web version of this article.)

To further explore the mechanisms of CS-induced BMSCs death, we conducted transcriptome RNA-sequencing of femurs from mice exposed to CS for 8 weeks and controls. A total of 5552 differentially expressed genes were discovered, including 2593 up-regulated genes and 2959 down-regulated genes (Fig. 3A and B), suggesting that CS exposure exerted a considerable effect on bone tissue. We further performed bioinformatics analysis of the differential genes and found that CS could inhibit the expression of a series of osteogenesis-related genes. Notably, the most significant change was observed in OCN, suggesting that osteoblast differentiation was potently inhibited by CS (Fig. 3C). Reactome annotations analysis showed that CS exerted the greatest effect on the immune system-related functions of bone tissue, suggesting that CS exposure significantly activated the inflammatory response of skeletal cells (Fig. 3D). To further explore the forms of cell death caused by CS in bone tissue, we performed enrichment analysis of differential genes with functional annotations for programmed cell death. We found that the “pyroptosis” pathway was most activated by CS among different types of programmed cell death (Fig. 3E). Collectively, our *in vivo* and *in vitro* results suggest that CS as a xenogeneic inflammatory stimulus might induce BMSCs pyroptosis, which may be a key mechanism for its inhibition of osteoblast differentiation.

### 3.4 CS exposure activates pyroptosis signaling pathway in bone marrow

**Fig. 4:**
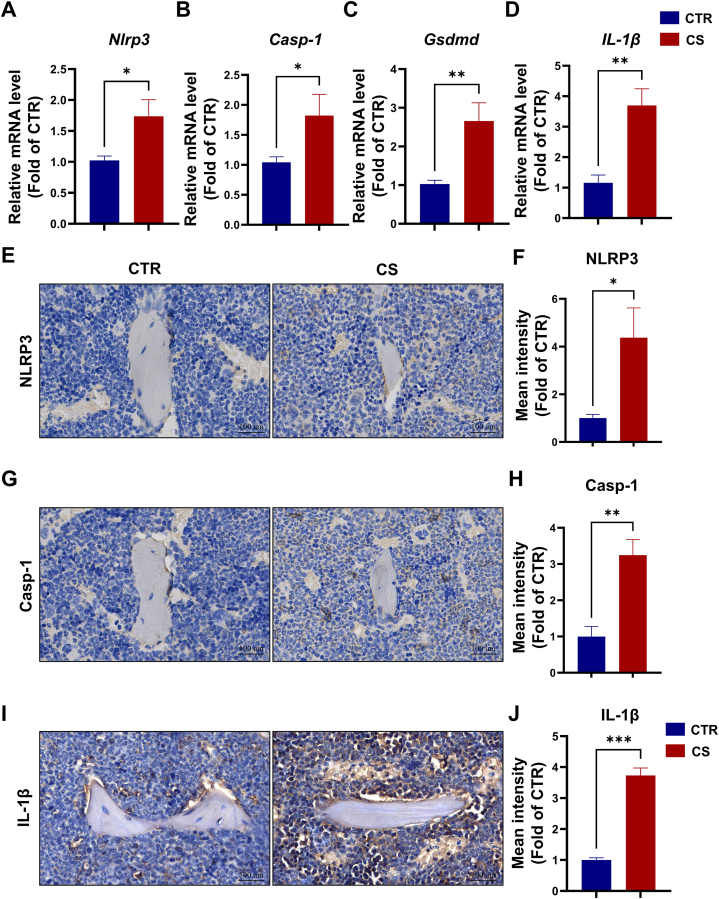
CS exposure activates pyroptosis signaling pathway in bone marrow. **(A**–**D)** The mRNA expression levels of pyroptosis-related genes (Nlrp3, Casp-1, Gsdmd and IL-1β) from mice exposed to CS for 8 weeks. **(E)** Representative immunohistochemical staining images for NLRP3 from mice in CTR and CS groups, scale bars: 100um. **(F)** Quantification of mean intensity for NLRP3, n = 6. **(G)** Representative immunohistochemical staining images for Casp-1 from mice in CTR and CS groups, scale bars: 100um. **(H)** Quantification of mean intensity for Casp-1, n = 6. **(I)** Representative immunohistochemical staining images for IL-1β from mice in CTR and CS groups scale bars: 100um. **(J)** Quantification of mean intensity for IL-1β, n = 3. *p < 0.05, **p < 0.01, or ***p < 0.001, respectively.

The canonical pathway of pyroptosis is that the stimulated inflammasome NLRP3 activates CASPASE-1, which causes GSDMD to cleave the cell membrane, ultimately leading to the secretion of inflammatory factors such as IL-1β. *In vivo*, the qRT-PCR results showed a significant increase in gene expression of Nlrp3, Caspase-1, Gsdmd, and IL-1β in the femur of CS-exposed mice (Fig. 4A–D). Immunohistochemical staining results of femur showed that protein levels of NLRP3, CASPASE-1 and IL-1β were significantly upregulated by CS exposure (Fig. 4E–J). These results further confirmed that the pyroptosis pathway was significantly activated by CS in bone tissue.

### 3.5 CSE induces NLRP3/caspase-1/GSDMD-mediated pyroptosis in BMSCs

**Fig. 5:**
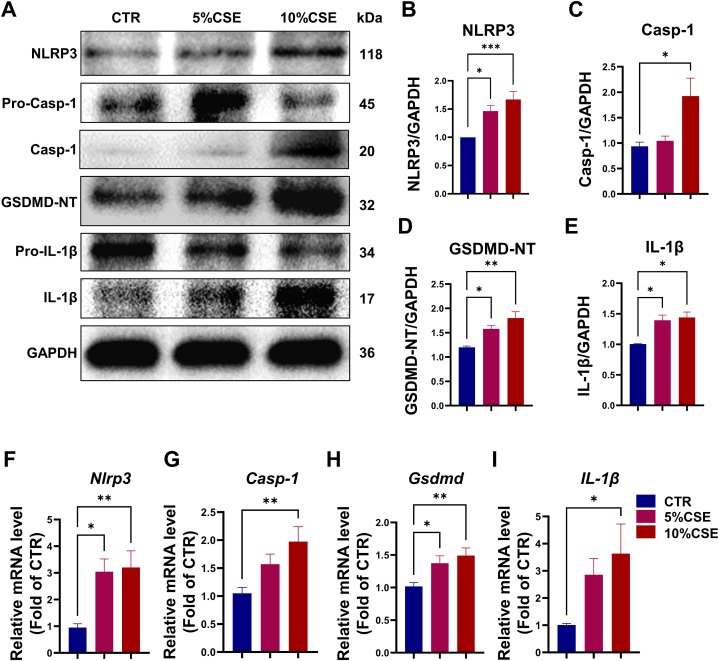
CSE induces NLRP3/caspase-1/GSDMD-mediated pyroptosis in BMSCs. **(A)** Representative Western blot images from BMSCs in CTR, 5 % CSE and 10 % CSE groups. **(B)** Quantification of intensity for NLRP3. (C) Quantification of intensity for Casp-1. **(D)** Quantification of intensity for GSDMD-NT. (E) Quantification of intensity for IL-1β. **(F–I)** The mRNA expression levels of pyroptosis-related genes (*Nlrp3*, *Casp-1*, *Gsdmd* and *IL-1β*) from BMSCs in CTR, 5 % CSE and 10 % CSE groups. *p < 0.05, **p < 0.01, ***p < 0.001, or ****p < 0.0001, respectively.

To further validate the mechanism by which CS causes BMSCs dysfunction by inducing pyroptosis, we further tested the effects of different concentrations of CSE on primary BMSCs *in vitro*. The western blotting results suggested that the protein levels of NLRP3, Caspase-1, GSDMD and IL-1β increased with increasing CSE concentrations in BMSCs (Fig. 5A–E). Consistently, the qRT-PCR results showed that the gene expression of pyroptosis-related genes significantly increased with CSE concentrations (Fig. 5F–I).

### 3.6 Pyroptosis inhibitor MCC950 attenuates CSE-induced deleterious effects on BMSCs

**Fig. 6:**
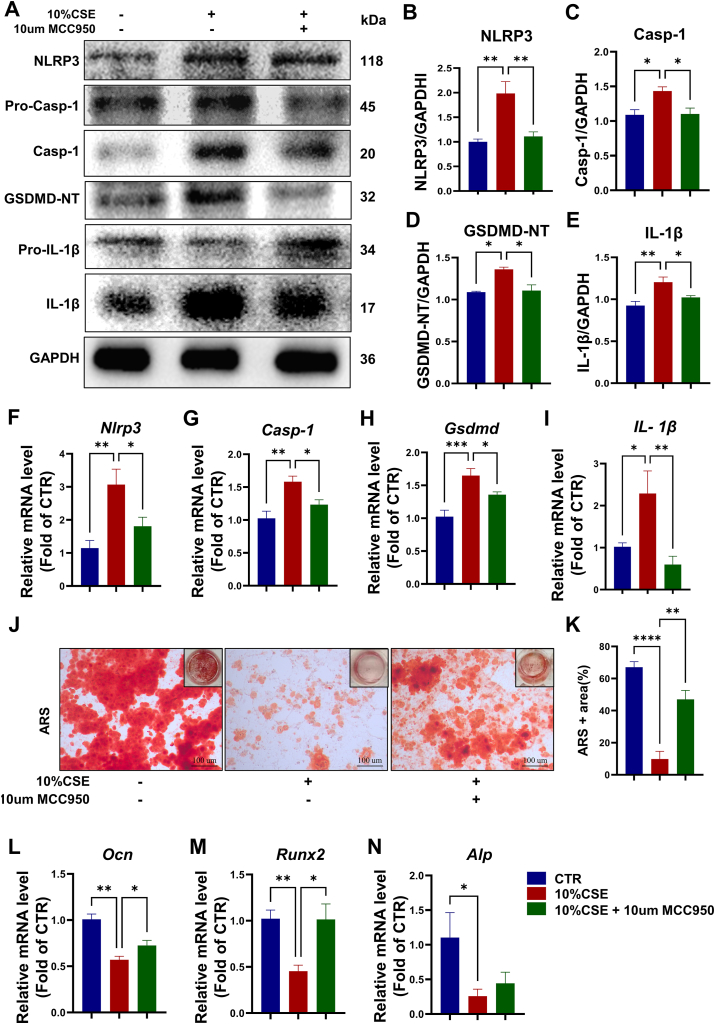
Pyroptosis inhibitor MCC950 attenuates CSE-induced deleterious effects on BMSCs. **(A)** Representative Western blot images from BMSCs in CTR, 10 % CSE and 10 % CSE + 10um MCC950 groups. **(B)** Quantification of intensity for NLRP3. (C) Quantification of intensity for Casp-1, n = 3. **(D)** Quantification of intensity for GSDMD-NT, n = 3. (E) Quantification of intensity for IL-1β, n = 6. **(F–I)** The mRNA expression levels of pyroptosis-related genes (*Nlrp3*, *Casp-1*, *Gsdmd* and *IL-1β*) from BMSCs CTR, 10 % CSE and 10 % CSE + 10um MCC950 groups. **(J)** Representative ARS staining images of BMSCs microscopy in CTR, 10 % CSE and 10 % CSE + 10um MCC950 groups, Scale = 100um. **(K)** Statistical analysis of ARS + area. n = 3–4. **(L**–**N)** The mRNA expression levels of osteogenesis-related genes (Ocn, Runx2 and Alp) from BMSCs CTR, 10 % CSE and 10 % CSE + 10um MCC950 groups. *p < 0.05, **p < 0.01, ***p < 0.001, or ****p < 0.0001, respectively.

MCC950, a selective inhibitor of the NLRP3-mediated pyroptosis pathway, was used to validate the activation of the canonical pyroptosis pathway and to treat pyroptosis-related disorders. To identify the key role of pyroptosis in CSE inhibition of osteogenic differentiation of BMSCs, we further tested the effects of MCC950 on CSE-treated BMSCs. As predicted, in BMSCs MCC950 effectively inhibited the CSE-activated Pyroptosis pathway at the gene and protein levels (Fig. 6A–I). Remarkably, MCC950 significantly increased the bone mineralization inhibited by CSE according to ARS staining (Fig. 6J and K). Moreover, the qRT-PCR results showed that MCC950 significantly up-regulated osteogenesis-related gene expression in CSE-treated BMSCs (Fig. 6L–N). Therefore, the pyroptosis inhibitor is effective in counteracting the detrimental effects of CSE on BMSCs.

## 4 Discussion

Smoking has been recognized as a predisposing factor for low bone mineral density, but basic research revealing the mechanisms of the effects of CS on bone metabolism is limited [27]. The mode, dose and duration of smoking exposure used are not standardized and the molecular mechanisms have not been explored in depth [8]. Most existing animal models of smoking exposure simulate inhalation of sidestream smoke, despite the fact that mainstream smoke is the primary form through which smoking impacts the human body. In the present study, we developed a device for animal mainstream smoke inhalation and established standardized smoke concentrations for exposure. Our observations revealed a time-dependent decline in bone density in mice exposed to CS, with 8 weeks of exposure routinely inducing a typical osteoporotic phenotype.

The basic pathogenesis of osteoporosis is enhanced OC-mediated bone resorption with a lack of compensatory OB-mediated bone formation [28]. In the mouse model of CS-induced osteoporosis, we found that CS significantly inhibited osteogenesis differentiation, as evidenced by reduced osteoblast numbers and suppression of osteogenesis-related genes. Considering osteoblasts are differentiated from BMSCs, we further tested the effect of various concentrations of CSE on mouse primary BMSCs *in vitro*. We found that CSE reduced cell viability, osteogenetic gene expression and bone formation capacity of mouse primary BMSCs in a dose-dependent manner. These results highlight the toxic effects of CS on BMSCs, suggesting its significant role in CS-induced bone loss.

Further investigation into the pathways and consequences of CS-induced death of BMSCs may contribute to elucidating the mechanisms through which CS adversely affects bone metabolism. We screened for genes that were significantly altered due to CS exposure by transcriptome sequencing of bone tissue. The analysis of these genes using bioinformatics techniques demonstrated that exposure to CS led to notable decreases in osteogenesis-related genes. Reactome annotations analysis showed that CS exposure most affected the expression of genes related to “immune system” pathways, emphasizing the potent inflammatory stimulation of the bone tissue by CS. Remarkably, the pathway associated with programmed cell death known as “pyroptosis” exhibited the most significant enrichment of differential genes in bone tissue exposed to CS. Hence, it was postulated that the exposure to CS is expected to trigger pyroptosis, resulting in detrimental effects on the process of osteogenic differentiation.

Pyroptosis is a novel mode of programmed cell death that is highly correlated with inflammatory responses and is strongly associated with the development of a variety of metabolic and spontaneous inflammatory diseases [29]. Canonical pyroptotic death is mediated by NLRP3 inflammasome assembly, which is accompanied by GSDMD cleavage and IL-1β and IL-18 release [30]. It has been found that CS exposure induces pyroptosis in multiple cells such as lung epithelial cells [31], peripheral blood macrophages [32], bladder epithelial cells [33] and cardiomyocytes [34]. However, the relationship between pyroptosis and osteoporosis-associated cells has not been established. In this study, we pioneered *in vivo* and *in vitro* to elucidate that CS exposure contributes to osteoporosis by inducing pyroptosis and subsequent inflammatory responses, as evidenced by a significant increase in the expression of NLPR3, Caspase-1, GSDMD, and IL-1β in the bone marrow of CS-exposed mouse and in CSE-treated BMSCs. Furthermore, to confirm that pyroptosis is an essential pathway for CS-impaired osteogenesis in BMSCs, the cellular pyroptosis inhibitor MCC950 was applied to CSE-treated BMSCs. Surprisingly, we observed that MCC950 effectively attenuated deleterious effects of CSE on BMSCs, as evidenced by improved cell viability, increased bone formation capacity and suppressed inflammatory factor secretion. As the knowledge of diseases caused by pyroptosis deepens, the use of pyroptosis inhibitors emerged as a possible therapeutic strategy for inflammation-associated diseases. Ma et al. proposed the neuroprotective role of Prussian blue nano-enzymes as pyroptosis inhibitors, providing a promising option for the treatment of Parkinson's disease [35]. Li et al. suggested that inhibition of NLRP3/GSDMD-mediated cellular pyroptosis may provide potential therapeutic benefit for astrocyte loss in the pathogenesis of depression [36]. Our study provided evidence to support the notion that the pyroptosis of BMSCs played a role in the impairment of osteogenesis caused by CS, thereby suggesting that targeting pyroptosis may hold promise as a preventive strategy for smokers in mitigating inflammatory osteoporosis. However, to fully demonstrate that inhibition of pyroptosis of BMSCs attenuates osteoporosis, adequate validation in in vivo experiments is required. Therefore, intervention with pyroptosis inhibitors in the CS-exposed mouse model as well as the establishment of BMSCs-specific gene editing mouse models are needed in future explorations.

In conclusion, the exposure to CS has been found to impede the process of osteogenetic differentiation and amplify the inflammatory response within the bone marrow, leading to significant bone loss. The activation of the pyroptosis pathway in bone marrow stromal cells (BMSCs) emerges as a pivotal mechanism underlying the development of osteoporosis induced by CS. The regulation of pyroptosis in BMSCs may provide a novel target for the prevention and treatment of osteoporosis.

## Ethics statement

Animal care and experimental procedures were supported by the Ethical Review Board at 10.13039/501100011790Xiangya Hospital of 10.13039/501100002822Central South University (approval no. 202112009).

## Funding statement

This study is supported by Hunan Province 10.13039/501100001809Natural Science Foundation of China (Grant Nos. 2022JJ40854), The Youth Science Foundation of 10.13039/501100011790Xiangya Hospital (Grant Nos. 2021Q07).

## Data availability statement

Data associated with the study has not been deposited into a publicly available repository and data will be made available on request.

## CRediT authorship contribution statement

**Guang Wang:** Writing – original draft, Visualization, Validation, Methodology, Formal analysis, Data curation, Conceptualization. **Hongming Li:** Validation, Methodology, Investigation, Formal analysis. **Xinyue Hu:** Methodology. **Yiyi Wang:** Methodology, Investigation. **Guoqiang Zhu:** Visualization, Methodology. **Hongliang Zhou:** Supervision, Software, Methodology. **Zilin Liang:** Methodology, Investigation, Data curation. **Zhenxing Wang:** Writing – review & editing. **Andreas Nuessler:** Writing – review & editing, Conceptualization. **Zhangyuan Lin:** Supervision, Resources. **Hui Xie:** Writing – review & editing, Resources, Project administration. **Sheng Zhu:** Writing – review & editing, Writing – original draft, Project administration, Funding acquisition, Conceptualization.

## Declaration of competing interest

We declare that we have no financial or personal relationships with other people or organizations that could inappropriately influence our work. There is no professional or other personal interest of any nature or kind in any product, service, and company that might be interpreted as exerting an influence on the content of this research.

## Figures and Tables

**Figure:**
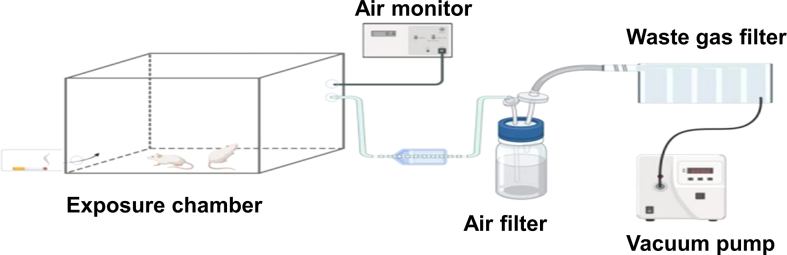
Supplemental Figure S1Animal mainstream smoke exposure device. Cigarette smoke was suctioned into the exposure chamber by a vacuum pump. The partial pressure of oxygen was maintained at 19%–20 % and carbon monoxide at 300 ppm ± 60 ppm. Air monitor was used to measure air parameters in the exposure chamber.

**Figure:**
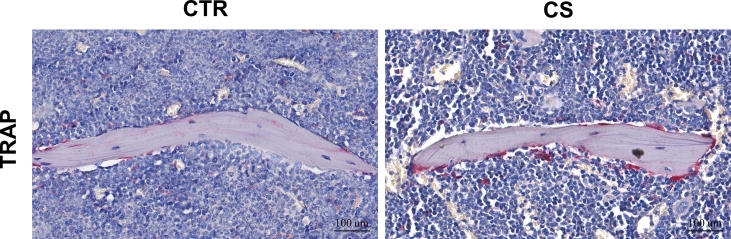
Supplemental Figure S2Representative TRAP-stained images in CTR and CS groups (8 week-exposure), scale bars:100 μm.
