# Supplementary material for: Exploring the relationship between pyroptosis and inflammatory bone loss: Evidence from a cigarette smoke-induced osteoporosis mouse model
Source: Heliyon. 2024 Aug 5;10(15):e35715. doi: 10.1016/j.heliyon.2024.e35715 (PMC11336831; doi:10.1016/j.heliyon.2024.e35715)

NLRP3

Full and uncropped western blot for Figure 5A
Lanes 1, 2, 3 are on the figure

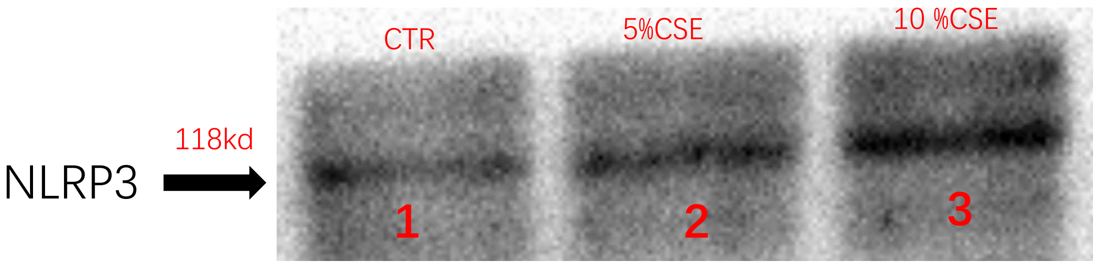

Full and uncropped western blot for Figure 6A
Lanes 1, 2, 3 are on the figure

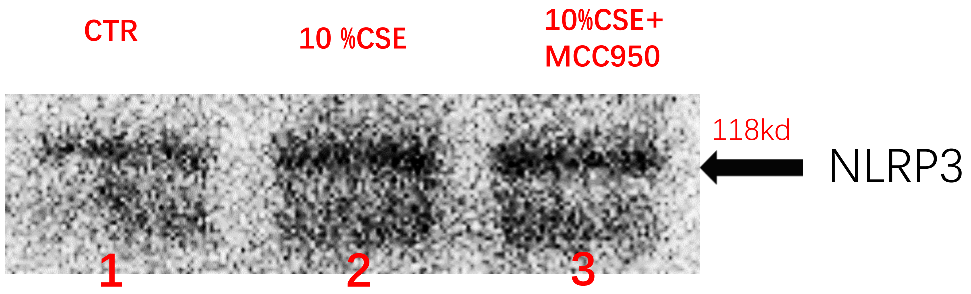

Casp-1

Full and uncropped western blot for Figure 5A
Lanes 1, 2, 3 are on the figure

Full and uncropped western blot for Figure 6A
Lanes 1, 2, 3 are on the figure

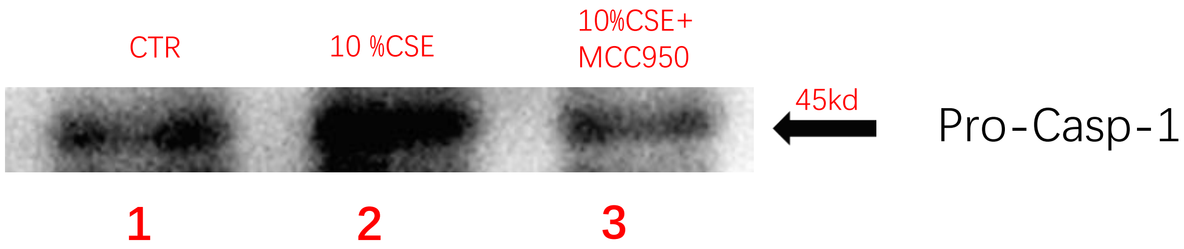

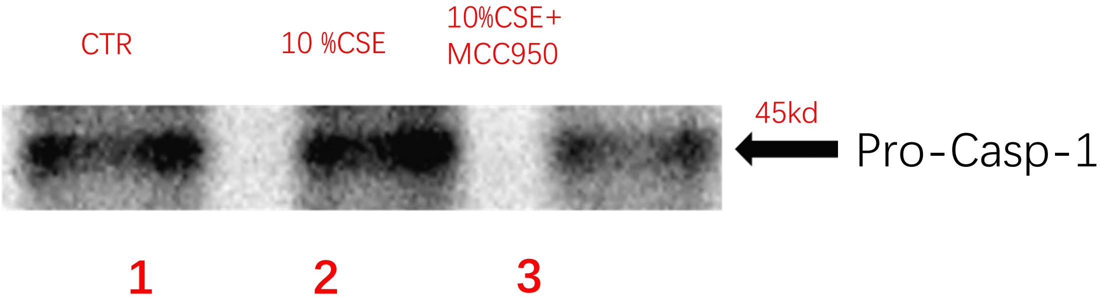

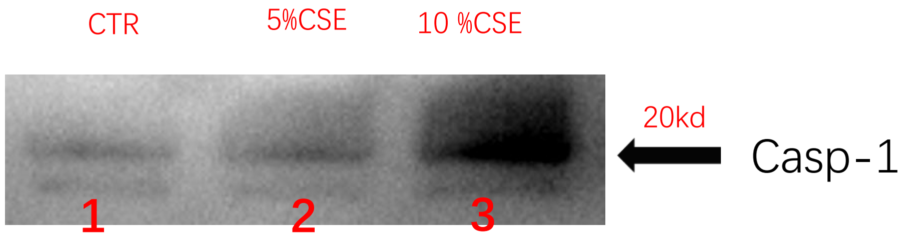

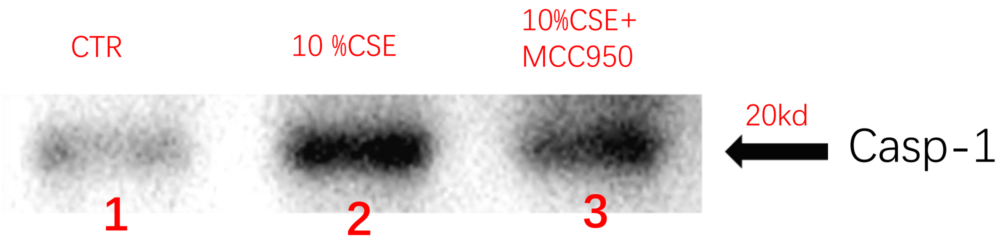

GSDMD-NT

Full and uncropped western blot for Figure 5A
Lanes 1, 2, 3 are on the figure

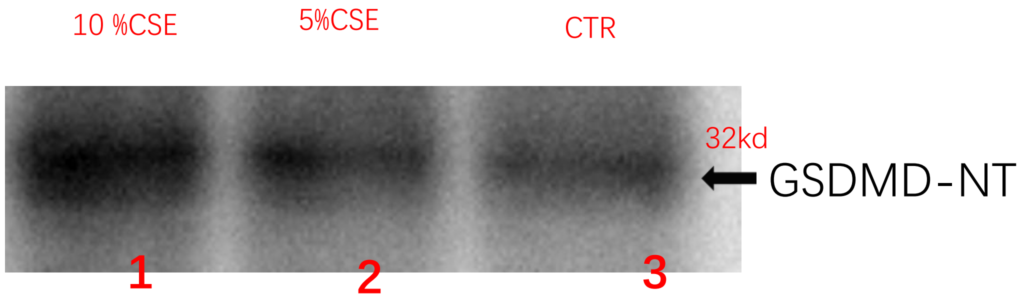

Full and uncropped western blot for Figure 6A
Lanes 1, 2, 3 are on the figure

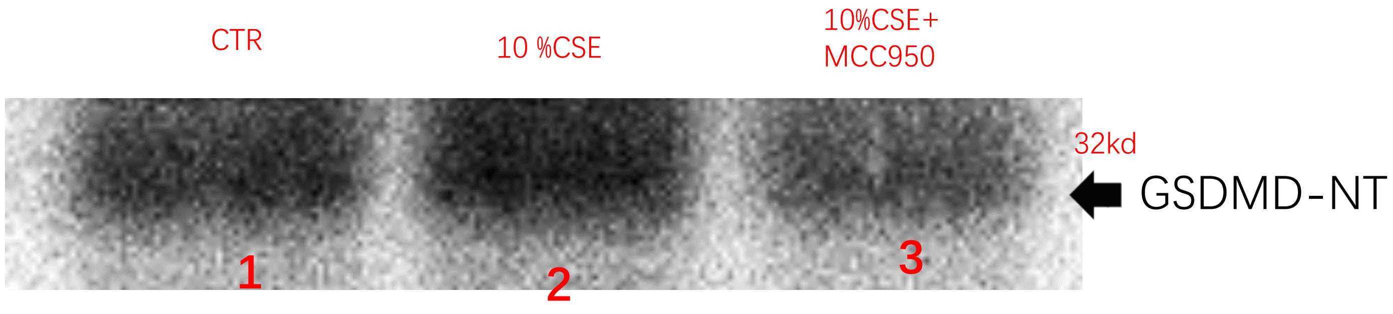

IL-1β

Full and uncropped western blot for Figure 5A
Lanes 1, 2, 3 are on the figure

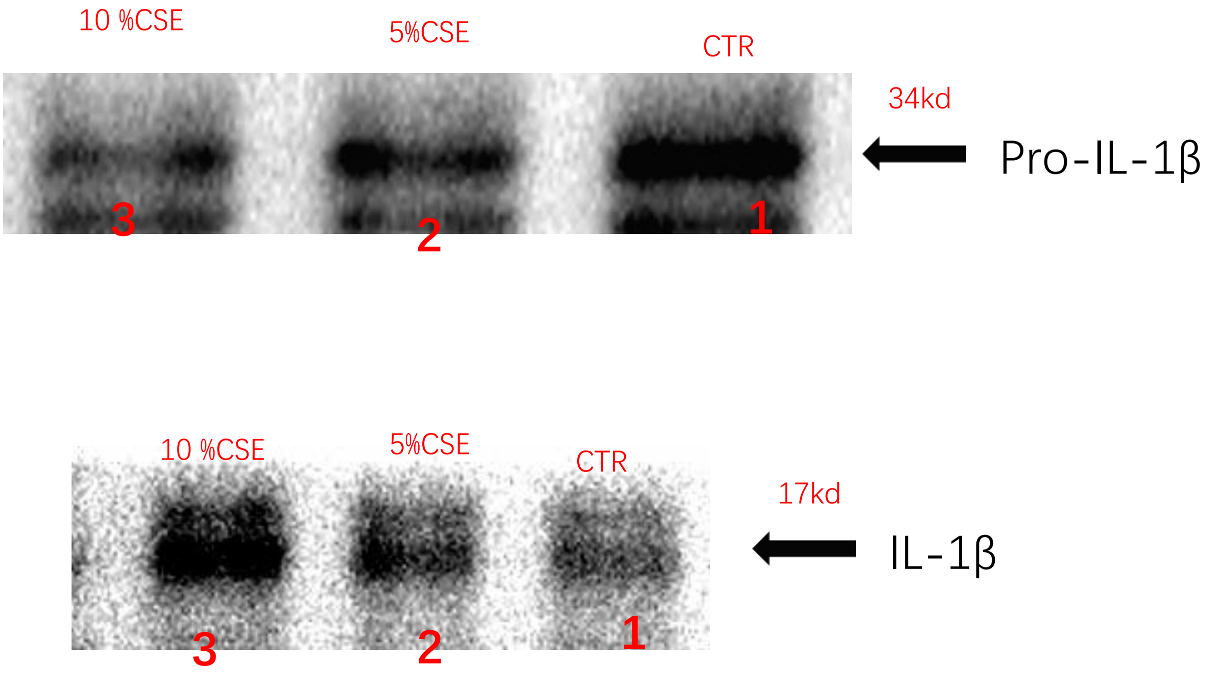

Full and uncropped western blot for Figure 6A
Lanes 1, 2, 3 are on the figure

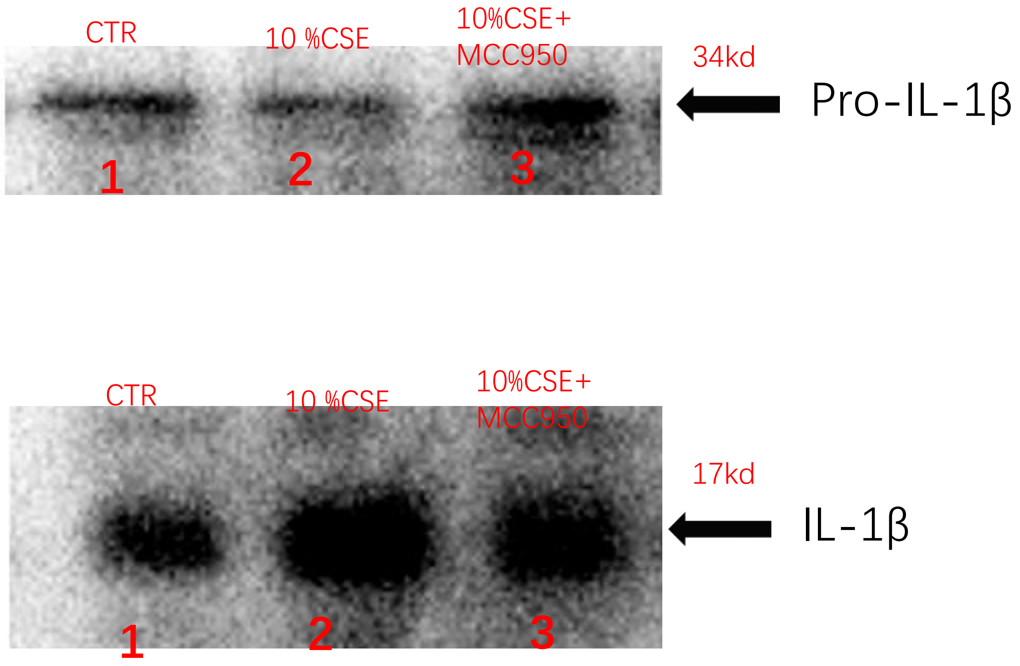

GAPDH

Full and uncropped western blot for Figure 5A
Lanes 1, 2, 3 are on the figure

GAPDH

36kd

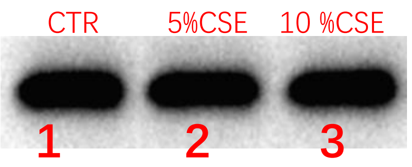

Full and uncropped western blot for Figure 6A
Lanes 1, 2, 3 are on the figure

GAPDH

36kd

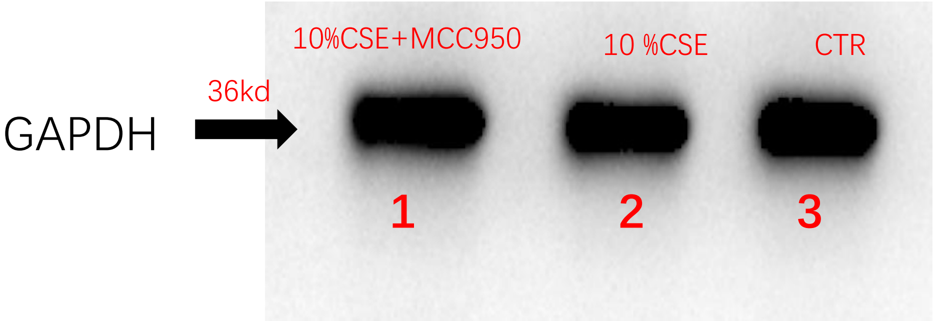

Supplement: Multimedia component 1 [file mmc1.pdf]
